# Impact of the Interaction Between Injury Mechanism and Intent on ICISS-Based Severity and Emergency Department Disposition: A Retrospective Study

**DOI:** 10.3390/healthcare14081036

**Published:** 2026-04-14

**Authors:** Ji-Hun Kang, Min-Seok Choi, Eun-Kyung Jung, Sung-Soo Choi, Seong-Ju Kim, Yun-Deok Jang

**Affiliations:** 1 Department of Emergency Medicine, Inje University Busan Paik Hospital, Busan 47392, Republic of Korea; mona@hanmail.net; 2 Department of Health Administration, Yeungnam University College, Daegu 42415, Republic of Korea; 3 Department of Paramedicine, Wonkwang University, Iksan 54538, Republic of Korea; 22jek@naver.com; 4 Department of Paramedicine, Gwangju University, Gwangju 61743, Republic of Korea; ranger898@naver.com; 5 Department of Paramedicine, Tongmyong University, Busan 48520, Republic of Korea

**Keywords:** emergency department, injury mechanism, patient transfer, suicide, accident

## Abstract

**Background/Objectives:** Injury mechanism and intent are key determinants of patient outcomes in the emergency department, yet their combined effects remain insufficiently understood. Emergency department disposition after injury may differ according to mechanism and intent and may be further influenced by specific mechanism–intent combinations. This study aimed to evaluate the associations of injury mechanism, intent, and their interaction with emergency department disposition and injury severity measured using the International Classification of Diseases-based Injury Severity Score (ICISS). **Methods:** We conducted a retrospective analysis of injury-related emergency department visits recorded between 1 January 2019 and 31 December 2023. Eligible visits included those with valid arrival and departure timestamps and complete disposition data; records with missing key variables or implausible length of stay were excluded. A total of 1,029,875 visits were analyzed. The primary outcome was emergency department disposition, categorized as discharge, admission, or transfer. Multinomial logistic regression was used to estimate relative risk ratios, with discharge as the reference category, and to derive predicted probabilities for selected mechanism–intent combinations. Injury severity was assessed using ICISS and modeled with injury mechanism, intent, their interaction, and prespecified covariates. **Results:** Of all visits, 69.9% resulted in discharge, 24.3% in admission, and 5.8% in transfer. Compared with traffic accidents, the highest likelihood of admission was observed in suffocation, drowning, and poisoning injuries. Transfer was more frequent in drowning, suffocation, penetrating injuries, and poisoning. Self-harm was associated with increased risks of both admission and transfer compared with unintentional injuries. Interaction analyses showed that certain combinations, particularly poisoning with self-harm and suffocation with self-harm, were associated with substantially higher risks than either factor alone. Predicted probabilities further highlighted high-risk combinations, including markedly elevated admission probabilities in self-harm-related poisoning and suffocation, and increased transfer probability in unintentional drowning. Injury mechanism, intent, and selected interactions were also significantly associated with ICISS-based injury severity. **Conclusions:** Injury mechanism and intent are independently associated with emergency department disposition and injury severity, with additional risk amplification observed for specific combinations. These findings suggest that mechanism–intent combinations may serve as clinically useful risk indicators in emergency department triage and decision-making, supporting improved risk stratification and system-level coordination.

## 1. Introduction

Injury is a major global health problem that causes substantial mortality and disability worldwide, including both unintentional injuries and intentional injuries such as violence and self-harm [1]. Injuries disproportionately affect adolescents and young adults and contribute to a large social and economic burden through premature death and long-term disability [1]. In clinical practice, a large proportion of injured patients enter the healthcare system through the emergency department, where early severity assessment, prioritization of care, and disposition decisions such as admission or interhospital transfer are made [2]. Therefore, improving risk stratification and disposition decision-making in the emergency department is essential to improve outcomes and optimize resource use [2].

In South Korea, the National Emergency Department Information System is a nationwide emergency care database that enables population-level analyses of emergency department utilization patterns, resource use, and outcomes [2].

Because it captures national-scale emergency department encounters, it provides a practical foundation to evaluate injury epidemiology and emergency care processes at the system level [2].

Recent Korean data also suggest that emergency department presentations related to self-harm and suicide attempts have shown meaningful changes over time, reinforcing the need to incorporate injury intent into injury research and emergency care planning [3,4].

Clinical trajectories and resource utilization among injured patients can vary substantially by injury mechanism and by intent [3,4]. In this study, injury intent refers to the underlying purpose or circumstance of the injury event, categorized as unintentional, self-harm/suicide attempt, assault/violence, or undetermined. For example, injuries categorized under similar mechanisms can have very different patterns of severity, comorbidity, clinical management needs, consultation requirements, and care pathways when the underlying intent differs [3]. Mechanism and intent may influence outcomes not only independently but also jointly through their combined effect, meaning that certain mechanism–intent combinations can be associated with particularly high risk [3,4].

Analyses that focus on only one of these factors may overlook clinically important interaction patterns and potentially understate or overstate risk in specific subgroups [3,4]. Standardized severity measurement is critical for interpreting findings and translating evidence into practice and policy [5]. The International Classification of Diseases–based Injury Severity Score is a diagnosis-code–based approach that estimates patient-level injury severity using survival risk ratios derived from large datasets, which makes it suitable for large administrative or registry-linked analyses [5]. ICISS differs from traditional severity scoring systems such as the Injury Severity Score (ISS) and Trauma and Injury Severity Score (TRISS), as it is derived from diagnosis-specific survival risk ratios and is particularly suitable for large administrative datasets.

In Korea, injury severity classification using ICD-based approaches has been actively discussed and updated to align with contemporary coding and outcomes, supporting their applicability in nationwide emergency department datasets [6,7]. Prior research has generally accumulated in two main directions [2,5]. First, nationwide emergency department database studies have described injury-related patterns and trends in emergency department visits and outcomes across population subgroups and time periods [2]. Second, methodological studies have evaluated and refined ICD-based injury severity metrics, including ICISS, to support large-scale severity adjustment and prediction [5,6]. These contributions have improved system-level understanding of emergency care and strengthened the evidence base for injury surveillance and quality improvement [2,5].

However, several gaps remain [3,4]. Many studies have examined either injury mechanism or intent separately, while fewer have explicitly tested whether the interaction between mechanism and intent influences severity and disposition outcomes [3,4]. In addition, descriptive approaches or coarse categorization of severity may lead to information loss and limit inference about the circumstances under which admission or transfer decisions are more likely [2,5]. As a result, evidence remains limited on which specific mechanism–intent combinations are associated with higher severity and higher likelihood of admission or transfer in nationwide emergency department data [3,4]. This study addresses these gaps using a retrospective observational design based on nationwide emergency department raw data [2]. We simultaneously consider injury mechanism and intent and explicitly model their interaction to quantify associations with injury severity and emergency department disposition outcomes, including admission and transfer [2,5]. By leveraging diagnosis-code-based severity scoring and a nationwide dataset, this study aims to identify high-risk combinations that are directly relevant to triage refinement, pathway design, and transfer system optimization [5,7].

The primary objective of this study was to evaluate whether the interaction between injury mechanism and intent is associated with emergency department disposition and ICISS-based injury severity.

## 2. Materials and Methods

### 2.1. Study Design and Data Source

This retrospective observational study used the National Emergency Department Information System (NEDIS) raw dataset covering 1 January 2019, to 31 December 2023. A total of 1,048,575 injury-related emergency department (ED) visit records were identified, and each ED visit was treated as the unit of analysis.

The primary outcome was ED disposition at the end of the ED encounter, categorized as discharge, admission, or transfer. Disposition was recategorized into a three-level outcome—discharge (reference category), admission, and transfer—and analyzed using multinomial logistic regression to estimate the effects of injury mechanism, intent, and their interaction on ED disposition. Results were reported as relative risk ratios with 95% confidence intervals. To enhance clinical interpretability, predicted probabilities and marginal effects were additionally derived for each injury mechanism–intent combination. The secondary outcome was injury severity quantified using the International Classification of Diseases–based Injury Severity Score (ICISS). ICISS15 was used as the primary continuous severity metric, and ICISS20 was used for sensitivity analyses. ICISS15 and ICISS20 differ in the number of diagnosis codes used to derive survival risk ratios, with ICISS20 incorporating a broader set of diagnostic codes to potentially improve the robustness of severity estimation. Because ICISS is a continuous measure bounded between 0 and 1 and may be skewed toward the upper bound, regression models were fitted with ICISS as the dependent variable and included injury mechanism, intent, their interaction term, and covariates. ICISS was modeled as a continuous variable using linear regression with robust standard errors. Sensitivity analyses using alternative modeling approaches yielded consistent results.

Robustness to distributional assumptions was evaluated using linear regression with robust standard errors and, in sensitivity analyses, fractional logit or beta regression. Interaction effects were assessed using statistical tests of the interaction term and likelihood ratio tests comparing models with and without the interaction term. This study used de-identified secondary data with no personally identifiable information and was granted an exemption by the Institutional Review Board (IRB No. 2026-01-023). The unit of analysis in this study was an individual emergency department visit; therefore, repeat visits by the same patient may have been included. Both pediatric and adult patients were analyzed together. Information on trauma center level was not available in the dataset.

### 2.2. Study Population

**Figure 1:**
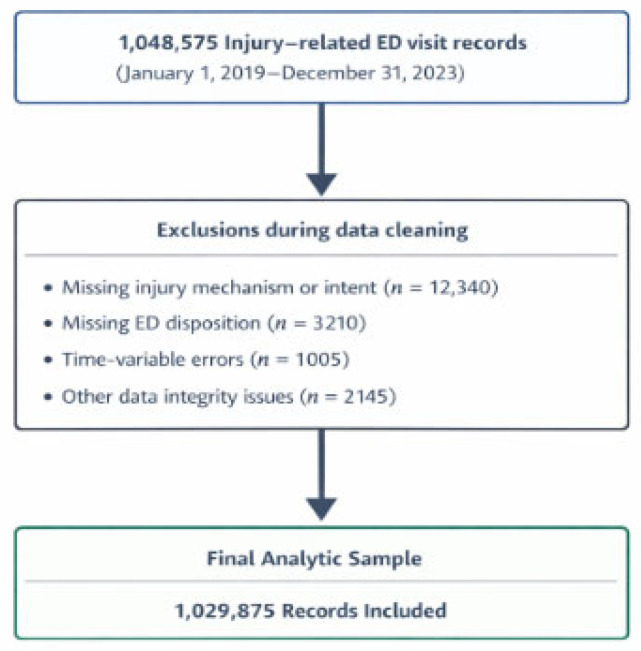
Study flow chart.

The study population comprised injury-related ED visits with available information on injury mechanism and intent and a recorded ED disposition outcome. Records were excluded during data cleaning if injury mechanism or intent was missing or unclassifiable, if ED disposition was missing, or if logical consistency could not be ensured due to clear input errors in key time variables or other core fields. For example, among 1,048,575 records, 12,340 were excluded due to missing injury mechanism or intent, 3210 due to missing disposition, 1005 due to time-variable errors, and 2145 due to other data-integrity issues, yielding 18,700 exclusions and a final analytic sample of 1,029,875 records (Figure 1).

### 2.3. Statistical Analysis

All analyses were conducted using R version 4.3.2 (R Foundation for Statistical Computing, Vienna, Austria; https://www.r-project.org/, accessed on 3 January 2026) and SAS version 9.4 (SAS Institute Inc., Cary, NC, USA; https://www.sas.com/, accessed on 3 January 2026). Two-sided tests were applied throughout, and statistical significance was defined as *p* < 0.05. Effect estimates were reported with 95% confidence intervals. For descriptive statistics, categorical variables were summarized as frequencies and percentages, and continuous variables were summarized as means and standard deviations for approximately normally distributed data or medians and interquartile ranges for non-normally distributed data. Continuous variables, including age and vital signs, were modeled as continuous variables without categorization. Covariates were selected based on clinical relevance and prior literature (a priori approach). When group comparisons were required, categorical variables were compared using the chi-square test or Fisher’s exact test, while continuous variables were compared using the independent-samples *t*-test. Three models were constructed for severity analysis (Model 1 included injury mechanism and intent, Model 2 additionally adjusted for prespecified covariates, Model 3 further included interaction terms between injury mechanism and intent). Prespecified covariates included age, sex, mode of arrival, transferred-in status, time of arrival, hospital characteristics, initial vital signs, and mental status. Interaction effects were assessed on the multiplicative scale through inclusion of interaction terms in the regression models.

## 3. Results

### 3.1. General Characteristics of Injury-Related Emergency Department Visits

A total of 1,029,875 injury-related emergency department (ED) visits were included in the analysis. The cohort comprised 519,875 males (50.5%) and 510,000 females (49.5%). The mean age was 47.8 ± 21.6 years; the most common age groups were 20–39 years (31.1%) and 40–59 years (30.1%), followed by 60–79 years (21.4%), ≥80 years (9.2%), and 0–19 years (8.3%).

Regarding arrival characteristics, 380,000 patients (36.9%) arrived by ambulance and 649,875 (63.1%) arrived as walk-ins/other modes. Transferred-in visits accounted for 120,000 cases (11.7%). ED arrival time was most frequently during the daytime (08:00–15:59; 54.4%), followed by evening (16:00–21:59; 27.2%) and night-time (22:00–07:59; 18.4%). Most patients were alert on arrival (89.3%), while 10.7% were categorized as non-alert.

Initial vital signs were as follows: systolic blood pressure 125.0 ± 22.0 mmHg, diastolic blood pressure 76.0 ± 14.0 mmHg, heart rate 88.0 ± 18.0 bpm, respiratory rate 18.0 ± 4.0 breaths/min, body temperature 36.7 ± 0.7 °C, and oxygen saturation 97.0 ± 3.0%.

The leading injury mechanisms were traffic accidents (25.2%) and falls/slips (23.3%), followed by blunt injuries (non-traffic) (14.6%), other/unspecified (15.5%), poisoning/intoxication (8.7%), and penetrating/cut/stab injuries (7.8%). Most injuries were unintentional (81.6%), whereas self-harm/suicide attempts (8.7%) and assault/violence (7.8%) constituted smaller proportions; 1.9% were undetermined. For ED disposition, 69.9% were discharged, 24.3% were admitted, and 5.8% were transferred. Injury severity, assessed using ICISS, was 0.972 ± 0.045 (ICISS15) and 0.968 ± 0.052 (ICISS20) (Table 1).

### 3.2. Association of Injury Mechanism, Intent, and Their Interaction with ED Disposition

In the multinomial logistic regression model, injury mechanism and intent were significantly associated with ED disposition. For admission vs. discharge, higher risks were observed for fall/slip (RRR = 1.18, 95% CI = 1.16–1.20), penetrating injury (RRR = 1.34, 95% CI = 1.30–1.38), thermal injury (RRR = 1.12, 95% CI = 1.07–1.18), poisoning/intoxication (RRR = 1.61, 95% CI = 1.56–1.66), suffocation/hanging/asphyxia (RRR = 2.45, 95% CI = 2.29–2.63), drowning/submersion (RRR = 2.10, 95% CI = 1.85–2.39), and other/unspecified (RRR = 1.05, 95% CI = 1.03–1.07) (all *p* < 0.001), whereas blunt (non-traffic) was associated with a lower risk (RRR = 0.96, 95% CI = 0.94–0.98; *p* < 0.001).

For transfer vs. discharge (reference mechanism: traffic accident), increased risks were found for fall/slip (RRR = 0.92, 95% CI = 0.89–0.95; *p* < 0.001) and blunt (non-traffic) (RRR = 0.88, 95% CI = 0.85–0.92; *p* < 0.001), indicating lower transfer risk, while penetrating injury (RRR = 1.22, 95% CI = 1.16–1.28; *p* < 0.001), poisoning/intoxication (RRR = 1.08, 95% CI = 1.01–1.16; *p* = 0.03), suffocation/hanging/asphyxia (RRR = 1.31, 95% CI = 1.14–1.51; *p* < 0.001), drowning/submersion (RRR = 1.52, 95% CI = 1.20–1.91; *p* < 0.001), and other/unspecified (RRR = 1.10, 95% CI = 1.06–1.14; *p* < 0.001) were associated with higher transfer risk; thermal injury was not significant (RRR = 0.97, 95% CI = 0.89–1.06; *p* = 0.48).

Compared with unintentional injuries, self-harm/suicide attempt was associated with higher risks of admission (RRR = 1.72, 95% CI = 1.67–1.77; *p* < 0.001) and transfer (RRR = 1.14, 95% CI = 1.06–1.22; *p* < 0.001). Assault/violence was also associated with admission (RRR = 1.09, 95% CI = 1.06–1.12; *p* < 0.001) and transfer (RRR = 1.28, 95% CI = 1.22–1.35; *p* < 0.001), and undetermined/unknown intent showed similar patterns (admission: RRR = 1.21, 95% CI = 1.15–1.27; *p* < 0.001; transfer: RRR = 1.34, 95% CI = 1.22–1.47; *p* < 0.001).

Several mechanism × intent interactions were significant: poisoning × self-harm (admission: RRR = 2.60, 95% CI = 2.45–2.75; *p* < 0.001; transfer: RRR = 1.22, 95% CI = 1.09–1.36; *p* < 0.001), fall/slip × self-harm (admission: RRR = 1.28, 95% CI = 1.20–1.37; *p* < 0.001; transfer: RRR = 0.95, 95% CI = 0.84–1.07; *p* = 0.40), penetrating × assault (admission: RRR = 1.52, 95% CI = 1.40–1.65; *p* < 0.001; transfer: RRR = 1.48, 95% CI = 1.32–1.66; *p* < 0.001), and suffocation × self-harm (admission: RRR = 1.90, 95% CI = 1.60–2.25; *p* < 0.001; transfer: RRR = 1.35, 95% CI = 1.05–1.75; *p* = 0.02). All models were adjusted for prespecified covariates (Table 2).

### 3.3. Association of Injury Mechanism, Intent, and Their Interaction with Injury Severity

Across the three models, injury mechanism and intent were associated with the outcome. In the fully adjusted model (Model 3; reference mechanism: traffic accident), fall/slip was positively associated with the outcome (β = 0.0012, 95% CI = 0.0006–0.0018; *p* < 0.001). Penetrating/cut/stab injuries showed a negative association (β = −0.0031, 95% CI = −0.0040–−0.0022; *p* < 0.001), as did poisoning/intoxication (β = −0.0048, 95% CI = −0.0057–−0.0039; *p* < 0.001), suffocation/hanging/asphyxia (β = −0.0065, 95% CI = −0.0084–−0.0046; *p* < 0.001), and drowning/submersion (β = −0.0042, 95% CI = −0.0070–−0.0014; *p* = 0.003). Other/unspecified mechanisms showed a small positive association (β = 0.0004, 95% CI = 0.0000–0.0009; *p* = 0.048). Blunt (non-traffic) (β = 0.0006, 95% CI = −0.0001–0.0013; *p* = 0.090) and burn/thermal injury (β = −0.0009, 95% CI = −0.0021–0.0003; *p* = 0.140) were not statistically significant in Model 3.

Regarding injury intent (reference: unintentional), self-harm/suicide attempt was consistently negatively associated with the outcome (Model 3: β = −0.0046, 95% CI = −0.0055–−0.0037; *p* < 0.001). Assault/violence also remained significant, although attenuated after adjustment (Model 3: β = −0.0010, 95% CI = −0.0018–−0.0002; *p* = 0.014), whereas undetermined/unknown intent was not significant in Model 3 (β = −0.0014, 95% CI = −0.0030–0.0002; *p* = 0.083).

In Model 3, selected mechanism-by-intent interaction terms were additionally examined. Poisoning × self-harm was significantly associated with a further decrease in the outcome (β = −0.0068, 95% CI = −0.0085–0.0051; *p* < 0.001). Suffocation × self-harm was also significant (β = −0.0040, 95% CI = −0.0069–0.0011; *p* = 0.007). In contrast, fall/slip × self-harm (β = −0.0016, 95% CI = −0.0033–0.0001; *p* = 0.064) and penetrating × assault (β = −0.0018, 95% CI = −0.0040–0.0004; *p* = 0.111) were not statistically significant. Model 1 was unadjusted, while Models 2–3 were adjusted for prespecified covariates (Table 3). These predicted probabilities should be interpreted in the context of baseline risks and overall case mix and do not independently account for all clinical severity factors.

### 3.4. Predicted Probabilities of ED Disposition by Injury Mechanism × Intent Combination

Predicted probabilities of ED disposition varied substantially by injury mechanism and intent. For unintentional traffic accidents, the predicted probabilities were discharge 70.0%, admission 25.0%, and transfer 5.0%. When intent was self-harm in traffic accidents, discharge decreased to 60.0% and admission increased to 34.0% (transfer 6.0%). For assault-related traffic accidents, transfer increased to 7.0% (discharge 67.0% and admission 26.0%).

Among falls/slips, unintentional injuries showed discharge 68.0%, admission 29.0%, and transfer 3.0%, whereas falls/slips with self-harm intent showed lower discharge (58.0%) and higher admission (39.0%) with transfer remaining at 3.0%. Blunt (non-traffic) unintentional injuries had a relatively high predicted discharge (73.0%) and lower admission (23.0%) and transfer (4.0%). Penetrating/cut/stab injuries were associated with higher transfer probabilities, particularly when intent was assault/violence (discharge 55.0%, admission 32.0%, and transfer 13.0%) compared with unintentional penetrating injuries (discharge 66.0%, admission 27.0%, and transfer 7.0%).

For poisoning/intoxication, unintentional cases showed discharge 63.0%, admission 33.0%, and transfer 4.0%, while poisoning with self-harm intent demonstrated the highest predicted admission (54.0%) and markedly reduced discharge (42.0%) (transfer 4.0%). The most severe pattern was observed for suffocation/hanging/asphyxia with self-harm intent, with discharge 30.0%, admission 62.0%, and transfer 8.0%. Drowning/submersion (unintentional) also showed a distinct disposition profile with elevated transfer (15.0%) alongside discharge (45.0%) and admission (40.0%). For other/unspecified injuries, unintentional cases showed the highest predicted discharge (75.0%) (admission 20.0% and transfer 5.0%), while undetermined/unknown intent was associated with higher transfer (8.0%) (discharge 70.0% and admission 22.0%) (Table 4).

## 4. Discussion

The purpose of this study was to examine, using nationwide injury-related emergency department visit data from 1 January 2019 to 31 December 2023, how injury mechanism and injury intent are associated with injury severity and emergency department disposition outcomes, with a particular focus on whether the interaction between injury mechanism and intent modifies these associations. These findings are consistent with previous studies reporting that injury mechanism and intent independently influence clinical outcomes; however, this study extends prior work by explicitly demonstrating interaction effects between these factors. Injuries impose a substantial global burden of mortality and disability, and diverse causes such as road traffic crashes, falls, drowning, burns, poisoning, self-harm, and violence frequently lead to emergency department visits and subsequent hospitalization; therefore, risk stratification at the emergency department stage is critical for both clinical care and policy [8,9]. In this study, injury mechanism and injury intent were each independently associated with injury severity and emergency department outcomes, and models incorporating the injury mechanism by intent interaction indicated that risk increased for specific mechanism–intent combinations. Although several associations were statistically significant, the magnitude of effect varied, and small relative risk ratios may have limited clinical significance despite statistical significance.

In addition, by classifying emergency department disposition into three categories, discharge, admission, and interhospital transfer, we were able to interpret admission and transfer as distinct outcomes. We also presented combination-specific predicted probabilities, providing clinically interpretable absolute risk information. Injury severity was assessed primarily using ICISS15, and robustness was evaluated through sensitivity analyses using ICISS20 and alternative modeling approaches under different distributional assumptions. ICISS is widely used for severity adjustment in large administrative datasets, as it derives patient-level severity from diagnosis-specific survival risk ratios, making it well suited for nationwide analyses such as the present study [10,11].

Although prior studies have accumulated important evidence describing the epidemiologic characteristics and clinical outcomes of injured emergency department patients, the scope of inference has often been limited by study design and analytic focus. Many studies have compared outcomes such as admission rate, transfer rate, mortality, or emergency department length of stay across injury mechanisms, or have explored risk factors focusing on specific mechanisms. While useful for demonstrating mechanism-specific differences, this approach may not adequately capture the fact that clinical trajectories and disposition decisions can differ by intent even within the same mechanism category. Research on self-harm or suicide attempts and violence-related injuries has also expanded, but these studies have frequently centered on characteristics of intent-defined groups or on selected outcomes, making it difficult to address combination-based questions such as which mechanisms are particularly high risk in specific intent contexts. Moreover, even in nationwide emergency department database studies, outcomes have often been simplified or disposition categories including transfer have been combined, limiting evidence that evaluates interhospital transfer as a distinct outcome. Although nationwide studies using NEDIS have demonstrated the utility of capturing national trends in emergency department utilization and disposition indicators such as admission and transfer, interaction-based analyses that integrate injury mechanism and intent in the injury field remain relatively limited [12,13]. Finally, although ICD-based severity measures such as ICISS have been used in prior work, analyses often remained at the level of describing differences by mechanism or intent, or did not explicitly model interactions, limiting systematic identification of high-risk combinations [10,11].

In this context, the present study is meaningful in that it addresses several limitations of the prior literature. First, by including injury mechanism and injury intent within the same model and explicitly specifying the injury mechanism by intent interaction term, we statistically tested combination effects. This approach helps identify risk amplification patterns that may be overlooked when focusing on mechanism or intent alone, and demonstrates that emergency department outcomes and injury severity can differ by intent even within the same injury mechanism. Second, by reconstructing disposition into three categories, discharge, admission, and transfer, and evaluating admission and transfer separately, this study provides evidence that is directly relevant to improving the emergency care delivery system. Because transfer is not merely a proxy for severity but reflects a combination of system factors including regional resources, hospital capability, and patient transport feasibility, analyzing transfer separately has important implications for system optimization. In addition, the nationwide scale of the dataset provided a sufficient sample size to improve the stability of estimates even for relatively uncommon combinations, and the presentation of combination-specific predicted probabilities offers absolute risk information that is readily interpretable in clinical settings. Finally, in severity analyses, we used ICISS15 as the primary metric while also applying ICISS20 and alternative regression approaches to demonstrate that findings were not unduly driven by a single metric or modeling choice, supporting the robustness of the results [10,11].

These findings have practical implications for emergency department triage, suggesting that specific mechanism–intent combinations should be considered as high-risk signals in clinical decision-making. Importantly, while mechanism–intent combinations provide useful risk signals, clinical decision-making remains primarily driven by patient-level severity and physiological status. Therefore, these combinations should be interpreted as complementary risk indicators rather than replacements for clinical judgment. For example, a low-severity traffic accident may not require intensive care, whereas a seemingly minor domestic injury may result in life-threatening conditions depending on bleeding or physiological compromise. First, in emergency department triage and standardized care pathway design, it is more reasonable to incorporate specific mechanism–intent combinations as high-risk signals rather than treating mechanism and intent as independent checklist items, and this supports the development of standardized operational guidance and practical checklists that define combination-based risk groups [8,9]. Second, to improve the reliability of data-driven policy, it is necessary to enhance the accuracy of injury mechanism and intent recording in the national emergency department database through standardized definitions and training, and to introduce quality indicators that monitor the proportion of unknown or unspecified entries at the institutional level. Third, from a transfer-system perspective, demonstrating differences in transfer probability by mechanism–intent combinations provides a basis for assessing regional imbalances in capabilities for severe trauma, poisoning, and critical care, and for optimizing transfer criteria and acceptance coordination; in regions where high-risk combinations concentrate, policy support is needed to strengthen transfer coordination functions that share real-time information on available beds and specialist resources. Fourth, absolute risk measures such as combination-specific predicted probabilities can be used not only to compare disposition patterns across hospitals or regions but also to develop performance monitoring and quality improvement metrics that account for differences in case mix; in the mid to long term, it may be reasonable to consider a phased application of combination-based risk-adjusted indicators in emergency care evaluation and quality improvement programs. Fifth, the identification of interaction patterns in severity analyses indicates that severity prediction is a multidimensional process shaped not only by ICD-based classification but also by intent, initial clinical status, and system-level factors; thus, future prediction modeling and clinical decision support tool design should incorporate interaction effects. In particular, for self-harm and violence-related injuries, there is a need to connect policy and clinical operations by institutionalizing continuity-of-care systems that link mental health services and community resources to reduce revisits and repeat events after disposition [8,9]. Incorporating these interaction-based risk patterns into triage protocols and interhospital transfer systems may improve resource allocation and patient outcomes.

This study has several limitations. First, as an administrative data-based study, misclassification of injury mechanism and intent is possible, and the presence of unknown or unspecified categories may introduce residual error. In addition, selection bias may have been introduced through the exclusion of records with missing or implausible data. Second, because the unit of analysis was an emergency department visit, repeated visits by the same patient may have been included, and patient-level correlation structures may not have been fully accounted for. Furthermore, clustering at the hospital level could not be explicitly modeled, which may have influenced variance estimation. Third, disposition outcomes and transfer decisions are strongly influenced by system-level factors such as bed availability, regional transfer networks, and institutional capability. Residual confounding may therefore remain due to unavailable variables, including comorbidities, insurance status, and trauma center level. Fourth, the multinomial logistic regression model assumes independence of irrelevant alternatives (IIA), which may not fully hold in the context of emergency department disposition, where admission and transfer decisions are interrelated. Finally, while ICISS is suitable for large-scale severity adjustment, it does not directly incorporate detailed clinical information such as physiologic severity, imaging findings, or surgical interventions, and severity estimates may vary according to coding accuracy and coding practices. Future studies should further stratify transfer outcomes according to underlying reasons and regional variations in healthcare resources.

## 5. Conclusions

Injury mechanism and intent were each independently associated with injury severity and emergency department disposition outcomes, and interaction patterns were identified in which risk was further amplified for specific combinations of mechanism and intent, suggesting that single-factor risk assessment may underestimate or overestimate risk in certain subgroups. In addition, by analyzing disposition outcomes as three categories, discharge, admission, and interhospital transfer, and presenting combination-specific predicted probabilities, this study provided clinically interpretable absolute risk information. These findings support the need for a combination-based risk assessment framework that integrates high-risk mechanism–intent combinations as distinct signals in emergency department triage and standardized care pathway design, rather than considering mechanism and intent only as independent checklist items. Furthermore, differences in transfer probability across combinations can be used to assess regional imbalances in capacity for severe trauma, poisoning, and critical care, and to optimize transfer criteria and acceptance coordination; combination-based prediction metrics may also contribute to the development of quality improvement monitoring and performance indicators that account for differences in case mix. Future research should externally validate prediction models based on mechanism–intent combinations and expand policy relevant evidence through multilevel analyses that incorporate hospital and regional level resource indicators to inform improvements in the emergency medical care delivery system. In particular, in South Korea, it would be reasonable to consider policy actions that strengthen standardization and quality assurance for recording injury mechanism and intent in the national emergency department database, progressively incorporate triage and transfer criteria reflecting high-risk mechanism–intent combinations into operational guidance for regional emergency medical centers and the severe trauma system, and enhance transfer coordination functions by linking real-time information on available regional resources with interhospital transfer decision making.

## Figures and Tables

**Table 1:** Baseline characteristics of injury-related emergency department visits (n = 1,029,875).

Variable	Category	n (%) or Mean ± SD
Sex	Female	510,000 (49.5)
	Male	519,875 (50.5)
Age (years)	Mean ± SD	47.8 ± 21.6
Age group	0–19	85,000 (8.3)
	20–39	320,000 (31.1)
	40–59	310,000 (30.1)
	60–79	220,000 (21.4)
	≥80	94,875 (9.2)
Mode of arrival	Ambulance	380,000 (36.9)
	Walk-in/Other	649,875 (63.1)
Transferred-in status	No	909,875 (88.3)
	Yes	120,000 (11.7)
Time of arrival	Day (08:00–15:59)	560,000 (54.4)
	Evening (16:00–21:59)	280,000 (27.2)
	Night (22:00–07:59)	189,875 (18.4)
Initial mental status	Alert	920,000 (89.3)
	Non-alert	109,875 (10.7)
Initial vital signs	Systolic blood pressure	125.0 ± 22.0
	Diastolic blood pressure	76.0 ± 14.0
	Heart rate	88.0 ± 18.0
	Respiratory rate	18.0 ± 4.0
	Body temperature	36.7 ± 0.7
	Oxygen saturation	97.0 ± 3.0
Injury mechanism	Traffic accident	260,000 (25.2)
	Fall/slip	240,000 (23.3)
	Blunt (non-traffic)	150,000 (14.6)
	Penetrating/cut/stab	80,000 (7.8)
	Burn/thermal injury	30,000 (2.9)
	Poisoning/intoxication	90,000 (8.7)
	Suffocation/hanging/asphyxia	15,000 (1.5)
	Drowning/submersion	5000 (0.5)
	Other/unspecified	159,875 (15.5)
Injury intent	Unintentional	840,000 (81.6)
	Self-harm/suicide attempt	90,000 (8.7)
	Assault/violence	80,000 (7.8)
	Undetermined/unknown	19,875 (1.9)
ED disposition	Discharge	720,000 (69.9)
	Admission	250,000 (24.3)
	Transfer	59,875 (5.8)
Injury severity	ICISS15	0.972 ± 0.045
	ICISS20	0.968 ± 0.052

Values are presented as n (%) for categorical variables and mean ± standard deviation (SD) for continuous variables. Counts were rounded to the nearest five for data presentation and confidentiality purposes in accordance with data use policies.

**Table 2:** Association of injury mechanism, intent, and their interaction with ED disposition.

Predictor	Admission vs. Discharge RRR (95% CI)	*p*-Value	Transfer vs. Discharge RRR (95% CI)	*p*-Value
Injury mechanism (reference: traffic accident)				
Fall/slip	1.18 (1.16–1.20)	<0.001	0.92 (0.89–0.95)	<0.001
Blunt (non-traffic)	0.96 (0.94–0.98)	<0.001	0.88 (0.85–0.92)	<0.001
Penetrating (involving cuts and stabbings)	1.34 (1.30–1.38)	<0.001	1.22 (1.16–1.28)	<0.001
Thermal injury (involving burns)	1.12 (1.07–1.18)	<0.001	0.97 (0.89–1.06)	0.48
Poisoning(involving intoxication)	1.61 (1.56–1.66)	<0.001	1.08 (1.01–1.16)	0.03
Suffocation/hanging/asphyxia	2.45 (2.29–2.63)	<0.001	1.31 (1.14–1.51)	<0.001
Drowning/submersion	2.10 (1.85–2.39)	<0.001	1.52 (1.20–1.91)	<0.001
Other/unspecified	1.05 (1.03–1.07)	<0.001	1.10 (1.06–1.14)	<0.001
Injury intent (reference: unintentional)				
Self-harm/suicide attempt	1.72 (1.67–1.77)	<0.001	1.14 (1.06–1.22)	<0.001
Assault/violence	1.09 (1.06–1.12)	<0.001	1.28 (1.22–1.35)	<0.001
Undetermined/unknown	1.21 (1.15–1.27)	<0.001	1.34 (1.22–1.47)	<0.001
Selected interaction terms (mechanism × intent)				
Poisoning × self-harm	2.60 (2.45–2.75)	<0.001	1.22 (1.09–1.36)	<0.001
Fall/slip × self-harm	1.28 (1.20–1.37)	<0.001	0.95 (0.84–1.07)	0.40
Penetrating × assault	1.52 (1.40–1.65)	<0.001	1.48 (1.32–1.66)	<0.001
Suffocation × self-harm	1.90 (1.60–2.25)	<0.001	1.35 (1.05–1.75)	0.02
Adjusted covariates	Yes		Yes	

Multinomial logistic regression was performed with discharge as the reference outcome category. Effect estimates are presented as relative risk ratios (RRRs) with 95% confidence intervals (CIs). Effect estimates are presented as relative risk ratios (RRRs), which are appropriate for multinomial logistic regression models. The fully adjusted model included covariates for sex, age, mode of arrival, transferred-in status, time of arrival, hospital characteristics, initial vital signs, and mental status. Interaction terms represent injury mechanism × injury intent effects. All *p*-values are two-sided, and statistical significance was defined as *p* < 0.05. ED denotes emergency department.

**Table 3:** Association of injury mechanism, intent, and their interaction with injury severity (ICISS15).

Predictor	Model 1 β (95% CI)	*p*-Value	Model 2 β (95% CI)	*p*-Value	Model 3 β (95% CI)	*p*-Value
Injury mechanism (reference: traffic accident)						
Fall/slip	0.0016 (0.0010–0.0022)	<0.001	0.0013(0.0007–0.0019)	<0.001	0.0012(0.0006–0.0018)	<0.001
Blunt (non-traffic)	0.0009 (0.0001–0.0017)	0.026	0.0007 (−0.0001–0.0015)	0.081	0.0006 (−0.0001–0.0013)	0.090
Penetrating/cut/stab	−0.0036 (−0.0046–−0.0026)	<0.001	−0.0033 (−0.0043–−0.0023)	<0.001	−0.0031 (−0.0040–0.0022)	<0.001
Burn/thermal injury	−0.0014 (−0.0028–0.0000)	0.051	−0.0011 (−0.0024–0.0002)	0.098	−0.0009 (−0.0021–0.0003)	0.140
Poisoning/intoxication	−0.0054 (−0.0064–−0.0044)	<0.001	−0.0050 (−0.0059–−0.0041)	<0.001	−0.0048 (−0.0057–−0.0039)	<0.001
Suffocation/hanging/asphyxia	−0.0072 (−0.0094–−0.0050)	<0.001	−0.0068 (−0.0089–−0.0047)	<0.001	−0.0065 (−0.0084–−0.0046)	<0.001
Drowning/submersion	−0.0051 (−0.0083–−0.0019)	0.002	−0.0045 (−0.0075–−0.0015)	0.004	−0.0042 (−0.0070–−0.0014)	0.003
Other/unspecified	0.0006 (0.0001–0.0011)	0.019	0.0005 (0.0000–0.0010)	0.052	0.0004 (0.0000–0.0009)	0.048
Injury intent (reference: unintentional)						
Self-harm/suicide attempt	−0.0053 (−0.0063–−0.0043)	<0.001	−0.0049 (−0.0058–−0.0040)	<0.001	−0.0046 (−0.0055–−0.0037)	<0.001
Assault/violence	−0.0016 (−0.0025–−0.0007)	<0.001	−0.0012 (−0.0020–−0.0004)	0.003	−0.0010 (−0.0018–−0.0002)	0.014
Undetermined/unknown	−0.0020 (−0.0038–−0.0002)	0.030	−0.0016 (−0.0032–0.0000)	0.052	−0.0014 (−0.0030–0.0002)	0.083
Selected interaction terms (mechanism × intent)						
Poisoning × self-harm	—	—	—	—	−0.0068 (−0.0085–−0.0051)	<0.001
Fall/slip × self-harm	—	—	—	—	−0.0016 (−0.0033–0.0001)	0.064
Penetrating × assault	—	—	—	—	−0.0018 (−0.0040–0.0004)	0.111
Suffocation × self-harm	—	—	—	—	−0.0040 (−0.0069–−0.0011)	0.007
Adjusted covariates	No		Yes		Yes	

Values are presented as regression coefficients (β) with 95% confidence intervals (CIs) for ICISS15, a continuous measure ranging from zero to one, where lower values indicate greater injury severity. Model 1 included injury mechanism and injury intent only (unadjusted). Model 2 was additionally adjusted for prespecified covariates, including sex, age, mode of arrival, transferred-in status, time of arrival, hospital characteristics, initial vital signs, and mental status. Model 3 further included selected injury mechanism × injury intent interaction terms. All *p*-values are two-sided, and statistical significance was defined as *p* < 0.05.

**Table 4:** Predicted probabilities of ED disposition by injury mechanism × intent combination.

Injury Mechanism	Injury Intent	Predicted Discharge %	Predicted Admission %	Predicted Transfer %
Traffic accident	Unintentional	70.0	25.0	5.0
Traffic accident	Self-harm/suicide attempt	60.0	34.0	6.0
Traffic accident	Assault/violence	67.0	26.0	7.0
Fall/slip	Unintentional	68.0	29.0	3.0
Fall/slip	Self-harm/suicide attempt	58.0	39.0	3.0
Blunt (non-traffic)	Unintentional	73.0	23.0	4.0
Penetrating/cut/stab	Unintentional	66.0	27.0	7.0
Penetrating/cut/stab	Assault/violence	55.0	32.0	13.0
Burn/thermal injury	Unintentional	72.0	25.0	3.0
Poisoning/intoxication	Unintentional	63.0	33.0	4.0
Poisoning/intoxication	Self-harm/suicide attempt	42.0	54.0	4.0
Suffocation/hanging/asphyxia	Self-harm/suicide attempt	30.0	62.0	8.0
Drowning/submersion	Unintentional	45.0	40.0	15.0
Other/unspecified	Unintentional	75.0	20.0	5.0
Other/unspecified	Undetermined/unknown	70.0	22.0	8.0

Predicted probabilities (percentages) of ED disposition (discharge, admission, and transfer) were derived from the fully adjusted multinomial logistic regression model. Probabilities were estimated with covariates held constant (e.g., at reference categories for categorical variables and at mean values for continuous variables). Percentages may not sum to exactly 100% due to rounding. ED denotes emergency department.

## Data Availability

No new data were created in this study. The data analyzed were obtained from the National Emergency Department Information System (NEDIS), operated by the National Emergency Medical Center, and are not publicly available due to privacy and ethical restrictions. The analysis code is available from the corresponding author (Y.-D.J.) upon reasonable request.

## Abbreviations

The following abbreviations are used in this manuscript:

ED	Emergency Department
NEDIS	National Emergency Department Information System
ICISS	International Classification of Diseases–based Injury Severity Score
ICISS15	International Classification of Diseases–based Injury Severity Score, 15-diagnosis Survival Risk Ratio Set
RRR	Relative Risk Ratio
OR	Odds Ratio
CI	Confidence Interval
SBP	Systolic Blood Pressure
DBP	Diastolic Blood Pressure
HR	Heart Rate
RR	Respiratory Rate
BT	Body Temperature
SpO_2_	Peripheral Oxygen Saturation
IRB	Institutional Review Board
